# Computation harvesting from nature dynamics for predicting wind speed and direction

**DOI:** 10.1371/journal.pone.0295649

**Published:** 2023-12-14

**Authors:** Takumi Aita, Hiroyasu Ando, Yuichi Katori

**Affiliations:** 1 Graduate School of Science and Technology, University of Tsukuba, Tsukuba, Japan; 2 Advanced Institute for Materials Research, Tohoku University, Sendai, Japan; 3 School of Systems Information Science, Future University of Hakodate, Hakodate, Japan; 4 Institute of Industrial Science, The University of Tokyo, Tokyo, Japan; Sunway University, MALAYSIA

## Abstract

Natural phenomena generate complex dynamics because of nonlinear interactions among their components. The dynamics can be exploited as a kind of computational resource. For example, in the framework of natural computation, various natural phenomena such as quantum mechanics and cellular dynamics are used to realize general purpose calculations or logical operations. In recent years, simple collection of such nature dynamics has become possible in a sensor-rich society. For example, images of plant movement that have been captured indirectly by a surveillance camera can be regarded as sensor outputs reflecting the state of the wind striking the plant. Herein, based on ideas of physical reservoir computing, we present a methodology for wind speed and direction estimation from naturally occurring sensors in movies. Then we demonstrate its effectiveness through experimentation. Specifically using the proposed methodology, we investigate the computational capability of the nature dynamics, revealing its high robustness and generalization performance for computation.

## Introduction

**Fig 1:**
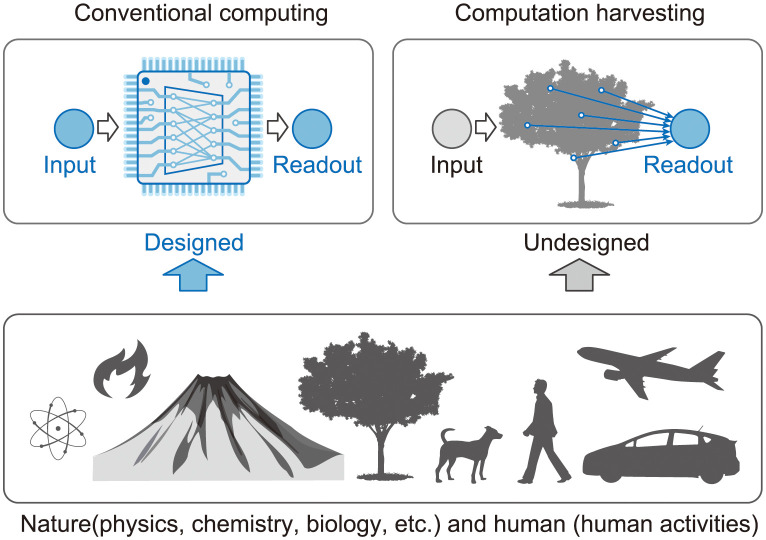
Computation harvesting. Schematic illustration of computation harvesting with reservoir computing (right) compared to the conventional one (left). The physical reservoir in the proposed method is not necessarily designed physically.

Conventional computing devices, ranging from digital computers to natural computing methods, are realized as a result of the precise design of natural phenomena in physical, chemical, and biological systems, as shown in Fig 1 and as explained hereinafter. The computational capabilities of such conventional computers are based on diverse physical quantities and dynamical phenomena inherent in nature. Specifically, recent publications include investigations pertaining to natural computations, with a particular emphasis on the utilization of physical media. This emphasis is particularly notable within the domains of biology and ecology [1–3]. However, such characteristics which are used as a basis of computation are not necessarily devised artificially: they are also available without design by human intervention. Those natural phenomena might be exploited as resources for computation if they could be extracted adequately without precise design. After discussing such unconventional natural computing with little human intervention from the perspective of computation harvesting from real-world phenomena, we validate the concept with demonstration by experimentation.

As described above, conventional computation with sophisticated algorithms such as machine learning is performed by physical phenomena inside computing devices, accompanied by elaborate design. In reality, physical phenomena in nature are capable of generating complex dynamics, but generally speaking, they cannot be used as they are for conventional computation as they are. Such non-designed natural dynamics if converted to computational processes could potentially support processes at low computational cost and with low energy expenditure. Specifically, natural phenomena involve complex interactions among their components and potentially generate rich patterns, which can be exploited to achieve some computing tasks and outcomes without the need for elaborate algorithms. Moreover, unintended computational capability can be acquired from nature. Based on these facts, computational processes can be extracted from natural phenomena: computation can be harvested.

First, the computation harvesting concept must be defined. If the target natural phenomena in the environment are not complicated, then one can model and simulate the phenomena mathematically using digital computers. However, generally speaking, most natural phenomena are so complex that they cannot be readily modeled. Accordingly, we assume that natural phenomena and their environment are regarded as a natural computer generating data and performance of the desired computation with the data. Practically speaking, the methods for extracting useful information obtained by sensing the real world and combining them for computation are crucially important. We designate the process of sensing dynamics in undesigned nature and the use of its information for computation as “computation harvesting”. Fig 1 presents a schematic illustration of the computation harvesting concept compared with conventional computing. Conventional computing systems are designed to be used with input devices to the computing units and to be used with read out sensors on the units. By contrast, in the proposed framework, computing units are not designed for computation, except for read out processes.

Among related works are several methods for extracting information from high-dimensional dynamical systems in nature. For example, natural computing is a popular methodology for unconventional computation with dynamics in natural phenomena such as cellular dynamics [4, 5], quantum mechanics [6, 7], and optical dynamics [8]. Recently, reservoir computing, which originates from recurrent neural network (RNN) based deep learning, is very popular in terms of computation with nonlinear dynamical systems [9–11]. Specifically, the reservoir corresponding to RNN can be replaced with physical devices such as soft materials, laser systems, and electric circuits [12]. They are regarded as constituting a natural computing system. Reservoir computing is applied to time series prediction, speech, and image recognition, and so on [13]. For any mode of conventional natural computing, precise design of computing devices is necessary. By contrast, computation harvesting is aimed at reducing the artificial design to the greatest extent possible, together with maximization of the computational capability inherent in nature.

To implement the concept of computation harvesting in natural phenomena, we specifically examine the information collected from sensors in the installed infrastructure. Recent advancements in the sensor technologies and the widespread use of information communication technologies (ICTs) have made it possible to acquire very large amounts of real-world information [14]. So-called Internet of Things (IoT) technologies can collect huge amounts of data. In reality, however, most of the data are wasted. For example, surveillance cameras use the information they acquire only in emergencies, but they can continue to collect the information they record at all times. Therefore, for this study, we specifically examine the use of the information which can be captured collaterally from the sensors. As a simple experiment, we can demonstrate the possibility of estimating wind direction and strength from plant movements captured by a camera. Similarly, it was demonstrated that a camera can capture the vibration of plants according to sound waves, making it possible to decode the original sounds from the movie [15]. We regard the plant motion as a computational resource: the extracted time series obtained from the motion of the feature points on the plant are used as computing units. They are processing information about the wind against the plants. Although the basic idea of the proposed method for wind classification is similar to that used for physical reservoir computing, these computing units need not be designed for the target computation. Stated simply, the physical plant is used as it is without sensing devices on it. From natural motion, the feature point dynamics in the movie can be captured. Then, the time series at the feature points is regarded as output against the wind input which is processed by the plant. The feature points extracted from the movie are regarded as computing units on the plant. Then the readout weights are learned as a linear combination of the time series of the motion on the plant to classify the wind speed and direction. The learning algorithm is based on the reservoir computing. The following sections introduce detailed methods and results of classification obtained for test cases when using the proposed framework.

## Methods

As explained in the introduction, computation harvesting is possible when combining a set of time series obtained from nature dynamics. A set of time series can be defined as *X*, with the target time series as *y*. For example, the time series prediction of *y* can be done by a linear combination of the components in *X*. The predicted *y* is described as $\tilde{y}=W_{out}X$, where *W*_*out*_ is learned from a teacher signal *y* and *X*. As described herein, we specifically examine a prediction task for wind speed and direction as *y* from the motion of a plant as *X*.

### Subject

For the analyses conducted for this study, we used a time series of leaf and stem movements extracted from video images rather than physical sensors. For this experiment, we used *Monstera* spp. to record movies with resolution of 1920 × 1080 pixels while changing the direction and speed of the wind blowing on the plants with a circulating fan (YAR-AD237; Yamazen Corp.). For 6-class (24-class) classification, the wind direction was recorded in two (eight) directions with an interval of 180 (45) degrees around the plants. The wind speed was recorded at three levels (level 1, 4, and 7) at 60 FPS. With the experimental setup, we used the circulating fan of 23 cm diameter, which had a DC motor and three blades. This fan offers the flexibility of adjusting the air volume to eight distinct levels.

### Feature detection

To reduce the video size, a 3×3 max pooling filter R was applied to each red, green, and blue (RGB) color space. This reduction decreased the video resolution from 1920 × 1080 pixels to 640 × 360 pixels.

$$\begin{array}{c} x_{i,j}^{\prime }=\underset{\left(s,t\right)\in R_{i,j}}{\text{max}}\;x_{s,t}. \end{array}$$ (1)

From this video, we detect feature points for tracking the plant sway. A popular method for corner detection is to compute a corner response function (*R*). This method is based on Moverac’s method [16], which calculates the sum of squared differences (*SSD*) between a patch that is extracted from a part of the image and a patch that is moved slightly. Harris [17] improved upon this method and considered computation of the derivative instead of *SSD* between patches. Using Taylor expansion, he approximated the *SSD* by a derivative. The approximation is

$$\begin{array}{c} SSD\left(\Delta x,\Delta y\right)\approx \left(\Delta x\;\Delta y\right)\;A(\begin{array}{c} \Delta x\\ \Delta y \end{array}), \end{array}$$ (2)

where Δ*x* and Δ*y* respectively denote the movement distances of feature points in the directions of *x* and *y*. Also, *A* is called the structure tensor, as

$$A=[\begin{array}{cc} \left\langle I_{x}^{2}\right\rangle & \left\langle I_{x}I_{y}\right\rangle \\ \left\langle I_{x}I_{y}\right\rangle & \left\langle I_{y}^{2}\right\rangle \end{array}].$$

Here, *I* represents the luminance of image converted from RGB color; *I*_*x*_ and *I*_*y*_ respectively denote the partial derivatives of *I* with respect to *x* and *y* direction. When the SSD is large, *A* has two positive and large eigenvalues. Therefore there are corners: when the value of the corner response is large, the feature point can be detected. Also, *R* is

$$\begin{array}{c} R=\left|A\right|-k{\left(traceA\right)}^{2}=\lambda_{1}\lambda_{2}-k{\left(\lambda_{1}+\lambda_{2}\right)}^{2}, \end{array}$$ (3)

where λ_1_ and λ_2_ are the eigenvalues of *A*. To track objects more stably, Shi and Tomasi [18] replaced Eq 3 with

$$\begin{array}{c} R=\text{min}\;(\lambda_{1},\lambda_{2}). \end{array}$$ (4)

For this study, we detected the feature points according to the method described by Shi and Tomasi. First, we extract a 7 × 7 pixel patch from the image and calculate the value of *R*. Letting *R*_*max*_ be the largest among all *R*, then the threshold is λ = *R*_*max*_. A feature point is regarded as existing if *R* > λ. The distance between the feature points is at least 20 pixels.

### Object tracking

A gradient-based optical flow method [19] was used to track the feature points. When a tracked object *I* moves in the (Δ*x*, Δ*y*) direction after a Δ*t* step, then, the amount of luminance change can be approximated as shown below using Taylor expansion. *I*(*x*, *y*, *t*) is the luminance of the image at position (*x*, *y*) and time *t*.

$$\begin{array}{c} I\left(x,y,t\right)\approx I\left(x,y,t\right)+I_{x}\left(x,y,t\right)\Delta x+I_{y}\left(x,y,t\right)\Delta y+I_{t}\left(x,y,t\right)\Delta t. \end{array}$$ (5)

When tracking the same object, there is little change in luminance. The amount of change is zero. That is

$$\begin{array}{c} I_{x}\Delta x+I_{y}\Delta y+I_{t}\Delta t=0. \end{array}$$ (6)

Considering the first-order derivative with respect to time step Δ*t*, we can obtain the velocity vector ***v*** = (***v***_*x*_, ***v***_*y*_) as

$$\begin{array}{c} I_{x}\frac{\Delta x}{\Delta t}+I_{y}\frac{\Delta y}{\Delta t}+I_{t}=I_{x}v_{x}+I_{y}v_{y}+I_{t}=0. \end{array}$$ (7)

We also add a constraint using the Lucas–Kanade method [20] and compute the velocity vector at each time step. Assuming that the feature point and its surrounding points vary in the same way, we can form multiple equations, which can be replaced by

$$\begin{array}{c} Av=b, \end{array}$$ (8)

where

$$\begin{array}{c} A=(\begin{array}{cc} I_{x}\left(q_{1,1}\right) & I_{y}\left(q_{1,1}\right)\\ \vdots & \vdots \\ I_{x}\left(q_{n,n}\right) & I_{y}\left(q_{n,n}\right) \end{array}),\;b=(\begin{array}{c} -I_{t}\left(q_{1,1}\right)\\ \vdots \\ -I_{t}\left(q_{n,n}\right) \end{array}), \end{array}$$ (9)

where *q*_1,1_, ⋯, *q*_*n*,*n*_ = (*x*_1_, *y*_1_), ⋯, (*x*_*n*_, *y*_*n*_) are the coordinates of the points surrounding the feature point. Using our calculations, *n* × *n* = 15 × 15 pixels including feature points are extracted. Therefore, ***v*** is

$$\begin{array}{c} v={\left(A^{T}A\right)}^{-1}A^{T}b. \end{array}$$ (10)

To capture more global movements, a Gaussian pyramid is also used. This is a method of scaling down the original image by application of Gaussian blur: at level 1, the resolution is reduced to 1/4; at *m* level, it is reduced to 1/4^*m*^. We conducted the calculations using *m* = 3.

### Classification

A plant changes from its current state to the next state after being affected by wind as input. This mechanism of transition from the current state and input to the next state is similar to the update equation of the reservoir unit in reservoir computing.

Considering Echo State Network (ESN) as an example of such a method, the state of reservoir units at each time step is obtainable as

$$\begin{array}{c} x\left(n+1\right)=f(W_{\text{in}}u\left(n+1\right)+Wx\left(n\right)), \end{array}$$ (11)

where ***x***(*n*) stand for the states of reservoir units at time step *n*, ***u***(*n*) denotes the inputs, *W* expresses the weights for internal connection of reservoir units, *W*_in_ signifies an input weight matrix, and ***f*** represents the activation function such as a hyperbolic tangent. Assuming that the model has *M* reservoir units and *C* output units, then, in the original reservoir computing, such a model must be designed. However, when using our method, the observed plant state is used as ***x***. Time step *n* is equal to that of object tracking.

In the estimation process, the readout weights are used to obtain the output as

$$\begin{array}{c} y\left(n+1\right)=W_{\text{out}}\left(x\left(n+1\right)\right), \end{array}$$ (12)

where ***x***(*n* + 1) represents reservoir states. Plants can be regarded as a kind of physical reservoir: they oscillate because of wind input to their current states. Then we would be able to replace the time series of reservoir states with the plant oscillation dynamics. We calculated *W*_out_ using ridge regression as

$$\begin{array}{c} W_{\text{out}}=Y_{\text{target}}^{T}X{\left(X^{T}X+\alpha I\right)}^{-1}, \end{array}$$ (13)

where an *N* × *M* matrix *X* = (***x***(1), ⋯, ***x***(*N*))^T^ represents the time series of reservoir states. Also, *N* denotes the time steps. An *N* × *C* matrix *Y*_target_ is the time series of the training label. Also, *α* is the regularization parameter. We performed calculations with *α* = 10^6^. We classified the wind patterns using *W*_out_ and the time series data for testing input [21] ***u*** as

$$\begin{array}{c} \text{class}\left(u\left(n\right)\right)=\underset{k}{\text{arg}\;\text{max}}\left({\bar{y}}_{k}\left(n\right)\right), \end{array}$$ (14)

where $\bar{y}\left(n\right)=\left\{{\bar{y}}_{k}\left(n\right)\right\}=\frac{1}{\tau }\Sigma_{l=0}^{\tau -1}y\left(n-l\right)$ is *τ* step moving average. The value of $\bar{y}\left(n\right)$ is related with the likelihood of the wind class. The percentage of correctly classified steps is the accuracy rate, defined as

$$\begin{array}{c} accuracy=\frac{\sum_{n\in N}t{\left(n\right)}^{T}e\left(i\right)}{N}, \end{array}$$ (15)

where $i=\underset{n}{\text{arg}\;\text{max}}\;y\left(n\right)$ and where ***e***(*i*) is a *M*-dimensional one-hot vector in which *i*-th element is one and others are zeros. In addition, ***t***(*n*) is the one-hot vector for the target label vector. We also calculated the magnitude of the error using the cross-entropy error function [22]:

$$\begin{array}{c} L=-\frac{1}{N}\sum_{n\in N}\sum_{k\in C}t_{k}\left(n\right)\;\text{log}\;y_{k}\left(n\right). \end{array}$$ (16)

In this method, the readout weights are obtained using the Ridge regression, but we also examine the case in which multinomial logistic regression [23] is used to introduce nonlinearity. Multinomial logistic regression is an extension of logistic regression for binary classification to multiclass classification. This method estimates the class by the magnitude of the score adjusted so that the range of scores in each class is [0, 1] and so that the sum of the scores in all classes is 1. If one designates the function for obtaining the score as *g*, then the score for class *k* is expressed as follows when the *M*-dimensional feature ***x***(*i*) at a certain time step *i* is used as input.

$$\begin{array}{cc} g(i,k) & =\beta_{0,k}+\beta_{1,k}x_{1}\left(i\right)+\cdots +\beta_{M,k}x_{M}\left(i\right),\\ & =\beta_{k}^{T}x\left(i\right). \end{array}$$ (17)

For multinomial logistic regression, if the number of classes is *K*, then function *g* is expressed as the log-odds ratio for class *K*. Because the sum of the probabilities of all classes is 1, the probability of a class *k* is calculable as shown below.

$$\begin{array}{c} p_{k}=\frac{e^{\beta_{k}^{T}x}}{1+\sum_{j=1}^{K-1}e^{\beta_{j}^{T}x}}. \end{array}$$ (18)

### Verify the robustness of the weights *W*_out_

After we test whether the class can be estimated even when some of the trained readout weights are lost, we infer how many reservoir units become difficult to estimate when they are missing. First, we learn *W*_out_ using all 166 units detected on the plant. Next, we set the weights of the reservoir units to 0 in the order of their absolute values based on the magnitude of the weight corresponding to one class. Because the reservoir unit is the *x*-*y* co-ordinate of a feature point, the weights of the units belonging to the same feature point are also set to zero. Two units of the weights are removed from *W*_out_, which includes the weights of 166 units. Then, the correct answer rate is obtained when estimation is done again using the newly obtained weights. This procedure is repeated until 164 units are finally removed. We also compared the results of six methods of selecting a class as a criterion for removing units. Input data *X*′ constitute the standardized version of the original data *X* using the following equation.

$$\begin{array}{c} X^{\prime }=\frac{X-E[X]}{\sqrt{V[X]}} \end{array}$$ (19)

## Results

### Classification of six wind patterns

As for the first task of the proposed framework, we consider classification of both the wind direction and speed for six classes (two opposite directions and three speeds for each direction), as shown in the movies presented as S1 File. The principle of the Echo state network [9, 24] was exploited to classify the wind direction and speed, as described earlier in Methods. For convenience in explaining the proposed framework, we designate the dynamics of the feature points on the plant as a reservoir unit for convenience. The reservoir consists of a physically connected network of reservoir units on the plant. The readout weights from the reservoir state of each unit are used to classify the wind patterns. The time series was extracted by tracking the movement of the feature points using the optical flow with the gradient-based method, Lucas–Kanade method [20] and pyramid method [25].

**Fig 2:**
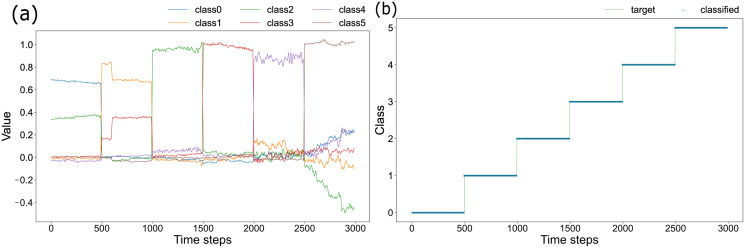
Six-class classification. Fig 2 (a) portrays the output obtained when the test data of time series from the plant are input for the trained readout weights with respect to classes separated according to color. The blue line shows Class 0, where the wind direction is to the left and the wind speed is low, with the largest value in the time step range from 0–500 steps compared to other lines representing other classes. Fig 2(b) shows the class which took the maximum value of the output shown in Fig 2 (a) at each time step. The green line is the correct label. The estimated classes are shown with blue crosses.

Because the plant is oscillating in response to the current wind inputs while taking over past states, we assume the possibility of considering the dynamics of feature points as the reservoir state in the Echo state network. Of the 92 feature points in total, 166 *x*-co-ordinates and *y*-co-ordinates of 83 points detected on the plants were regarded as reservoir units. The readout weights were calculated using the Ridge regression with those time series data. The weights obtained in such a way are presented in a related figure of S1 File. We classify six classes with respect to the test data using the trained readout weights. The input time series of test data are shown in a related figure of S1 File. As shown in Fig 2(a) and 2(b), the results enable us to classify plants accurately under six patterns of wind direction and speed based on time series data of all 83 feature points. The accuracy rate was 99.6%. It is noteworthy that Classes 0,2, and 4 respectively correspond to wind to the left direction with low, middle, and strong wind speeds. Also, Classes 1,3, and 5 respectively correspond to wind to the right direction with low, middle, and strong wind speeds. The value of the y-axis in Fig 2(a), which signifies the output generated by the inherent reservoir, can be interpreted as the class probability. The class characterized by the greatest probability is regarded as the estimated class.

**Fig 3:**
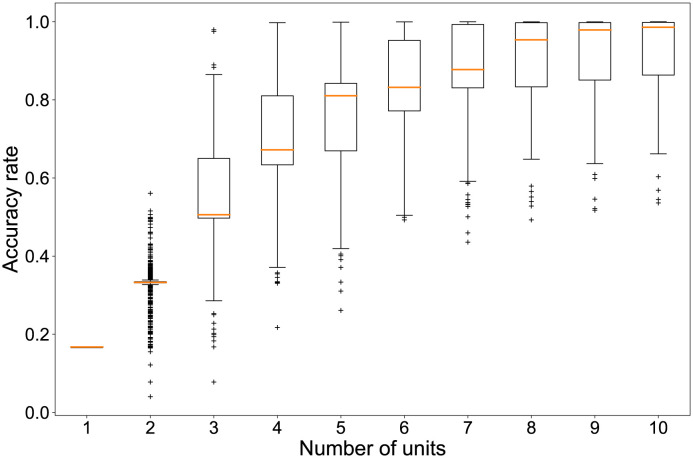
Required number of units for classification. Accuracy rates of six classifications depend on the number of sampled units. Boxplots show the distribution of the percentage of correct answers for randomly selected combinations of reservoir units. The vertical axis shows the percentage of correct answers. The horizontal axis shows the number of randomly selected units. For each number of units, we tried 1000 combinations of units and obtained the accuracy rate. Plus signs represent outliers.

Although the classification task described above is solvable using 166 reservoir units, we have confirmed the possibility of classifying the problem without using all of these units. In other words, classifying the wind patterns is possible even if we were unable to capture the entire plant or the time series of some units were lost. Fig 3 shows that a few units can be sufficient to identify the six classes. The vertical axis shows the percentage of correct answers for 1000 realizations of classification using only randomly selected reservoir units ranging from one to ten. According to these results, a time series from eight units can classify patterns with a reasonably high accuracy rate. In certain instances, meticulous scrutiny of a mere three points was adequate for achieving remarkably precise classification. Upon visual examination, the three featured points within the video manifested no discernible attributes associated with plant morphology. However, it is conceivable that a more intricate analysis might unveil the underlying biological characteristics which are crucially important for wind speed classification. This intriguing avenue warrants exploration in future research because ample scope for deliberation and investigation remains from both biological and ecological perspectives.

### Classification of 24 wind patterns

**Fig 4:**
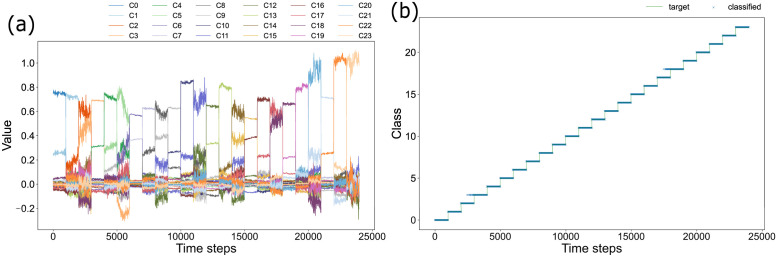
Classification for 24 classes. Fig 4(a) shows the output responding to 24 patterns of test time series for the trained readout weights. The 24 colors correspond to the outputs for all classes. Fig 4(b) shows the classification result obtained by taking the maximum of the outputs shown in Fig 4(a). The green line is the correct answer label. The estimated classes are shown with blue crosses. Crosses that are not on the green line correspond to incorrect classification.

The classification presented earlier included wind directions of two types of the plants: from the left and from the right. In addition, a classification task with three levels of wind speed and eight directions was performed to ascertain whether it can classify wind directions and speeds under numerous conditions. In the current case, 63 feature points were detected on the plants. All 126 reservoir units obtained were used for classification. The result is portrayed in Fig 4. The learned weights and test time series are presented in related figures of S1 File.

The task became more difficult because of the greater number of wind directions than those used for six-class classification, and because of the lower accuracy of estimation. However, when all the detected reservoir units were used, the accuracy rate was 99.1%, indicating good performance, even by a linear model for readout learning.

### Behaviours for untrained data

As the plant grows and as its structure changes in nature, the observed dynamics also change gradually. In our method, updating the readout weights used for estimation is easy because of low computation process costs. Nevertheless, it is noteworthy how well the proposed framework classifies untrained input time series when the weights are not re-trained for the not-trained inputs. The botanical growth evolves gradually over time, with distinct transformations in morphology occurring in response to seasonal changes. Consequently, the datasets we used are closely related to specific temporal snapshots representative of each season. Nevertheless, our algorithm possesses the adaptability to analyze and characterize plant states across all seasons. This adaptability arises from the rapidity of the learning phase, which is significantly shorter than the time scale governing plant growth. Therefore, it is entirely feasible to adjust algorithmic parameters in accordance with the prevailing season.

**Fig 5:**
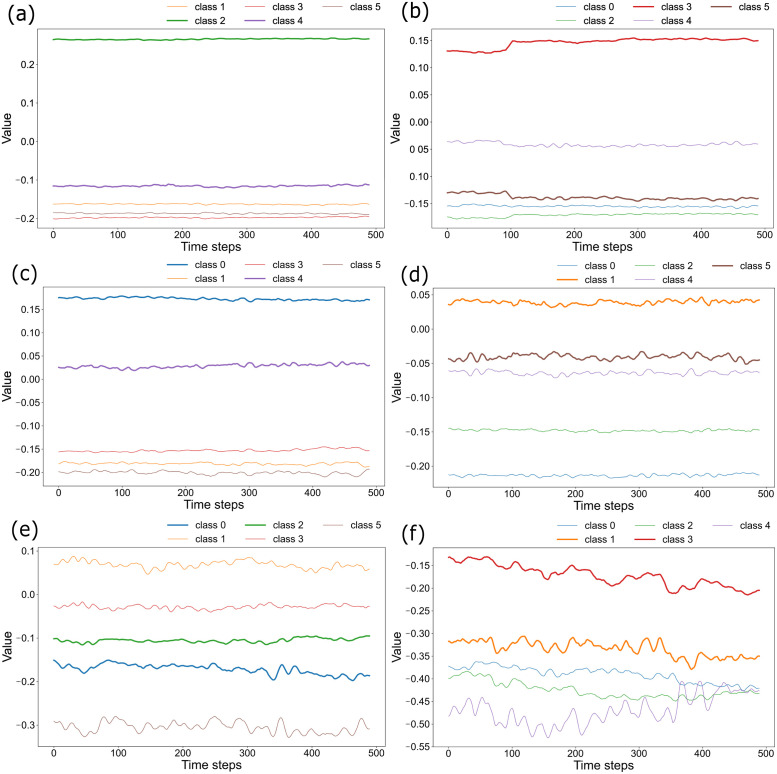
Generalization performance. Results of classification when one class out of six is lost. Fig 5(a) shows the values estimated when untrained test data of class 0 are input to the readout weights trained on *X*_*std*_ for classes 1–5. The output time series of classes 2 and 4, shown in green and purple, is larger than the others. These two classes have the same wind direction as that of class 0. The output of the class with the same wind direction as the input class is shown by the bold line in the figure. The output is also shown in Fig 5(b)–5(f). (b) Missing data of class 1. (c) Missing data of class 2. (d) Missing data of class 3. (e) Missing data of class 4. (f) Missing data of class 5.

First, we trained the readout weights using data from five classes, excluding any one of the six classes: then we investigated the input data from the untrained class by the obtained *W*_out_. Here, we train using standardized data *X*_*std*_ to avoid the influence of the magnitude of plant sway. The standardization process is presented in Eq 19. The results of output time series are presented in Fig 5.

The horizontal axis of Fig 5 shows the time steps. The vertical axis shows the magnitude of the output. Fig 5(a) presents the results obtained when class 0 data are input to the reservoir with regard to the readout weights trained with the data from which class 0 was removed. The outputs of classes 2 and 4, which are classes with the same wind direction as class 0, are greater than those of the others. Similarly, in most of the other cases in which one class is removed for training, the trained weights for the same wind direction as the input data tended to yield the greater output. This tendency suggests that the trained readout weights and learns some features related to the wind direction. It might be able to adapt to the use of untrained data.

### Robustness of weight

**Fig 6:**
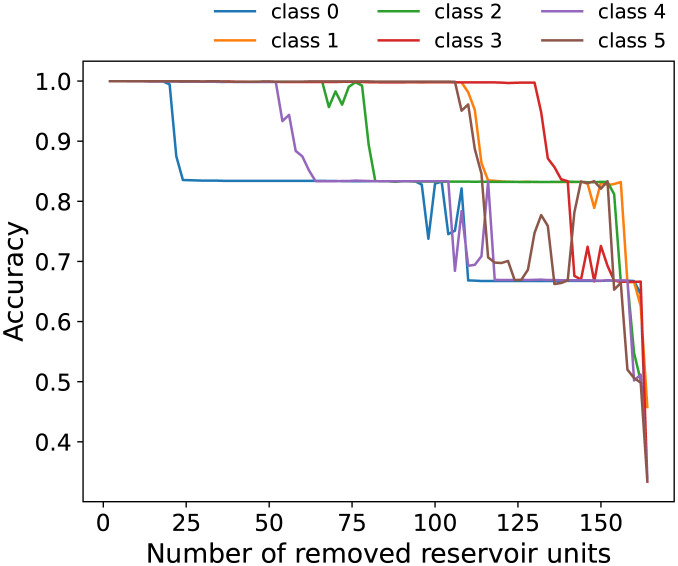
Measuring robustness. The classification accuracy depends on the number of removed units. The horizontal axis shows the number of reservoir units removed from the original readout weights trained by all units. The vertical axis shows the percentage of correct answers. Colors represent the classes used as the basis for removing the weights. For example, the blue line shows the percentage of correct answers when the units are removed in the order of the absolute value of the weights for the basis of class 0.

In practice, a case arises in which the reservoir units that were acquired initially can be lost because of changes in the external environment or because of malfunction of the imaging equipment. In such cases, some readout weights are not used for classification. The effects of lost units of the obtained readout weights on the classification accuracy were investigated. As shown in Fig 6, classification is performed when removing reservoir units by reducing the corresponding component of *W*_out_ in the order of increasing absolute value with respect to classes 0–5. For this reduction, operation means by which units of high importance for classifying each class are removed gradually. The readout weights were not re-trained when the unit was removed. Instead the response was observed when the already estimated component of *W*_out_ was set to zero. Fig 6 shows the dependence of the percentage of correct answers on the number of removed units. The input time series data are standardized as *X*_*std*_ by pre-processing. The reservoir units are removed one by one.

In six-class classification using all the reservoir units, the readout weights can adapt to the size of the coordinates, whether the input is raw *X* or standardized time series data *X*_*std*_. Consequently, it is possible to classify them correctly. However, the robustness test, in which some weights are set to zero, requires that the input time series should be standardized because differences among the variance of each time series cause incorrect classification.

As shown in Fig 6, the six classes were classified almost correctly using the readout weights trained from all 166 units of the detected time series. When we start removing feature points one by one, i.e., every two units of *W*_out_, the correct classification rate decreases in a step-wise fashion. Specifically examining the case of class 0, the percentage of correct answers decreased significantly when about 20 units were removed. Class 0 is not classified correctly in the first step down region, where the percentage of correct answers is constant and independent of the number of removed units. Class 0 cannot be classified because of removal of the large weights of this class. The next step down is at about 110 units of removal, for which class 2 and class 0 cannot be classified correctly. Those classes are with the same wind direction. The transition of the percentage of correct answers is sudden with constant percentages in a large area, which indicates that no difference exists in the classification results, even if the feature points are removed. The observation implies that learning with many reservoir units is redundant. In other words, it provides robustness to the readout weights.

### Data augmentation using time delays

It is desired that numerous reservoir units be extracted from the movie, but in reality, this extraction can be difficult in some cases. When the number of units is obtained insufficiently, the performance can be improved by pseudo-increasing the time series data. In a case where only a few reservoir units are available, we examined the augmentation of time series data using time delays to improve the classification accuracy. As a test case, three units were selected randomly from the 166 units for training data *X*(*t*). In addition to *X*(*t*), *τ* step-delayed time series were considered: *X*′(*t*) = [*X*(*t*), *X*(*t* + *τ*), ⋯, *X*(*t* + *dτ*)](*d* = 1, ⋯, 20).

**Fig 7:**
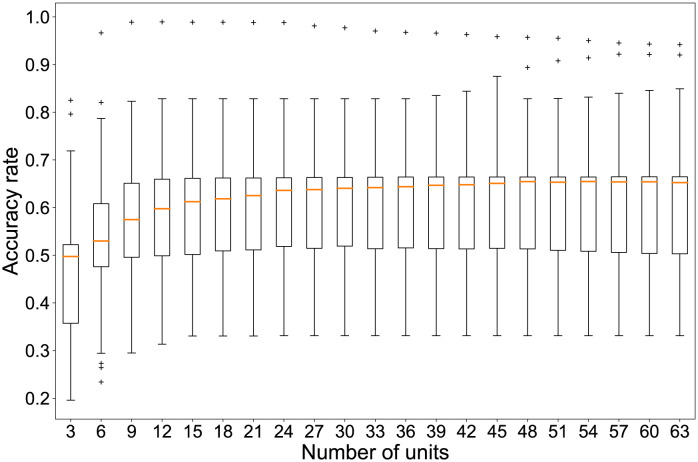
Effects of unit augmentation using time delay. Boxplots show the distribution of the accuracy for a randomly selected combination of reservoir units for 122 patterns with three units. The horizontal axis shows the number of augmented units. The vertical axis shows the accuracy. The number of units increases as *X*′(*t*) = [*X*(*t*), *X*(*t* + *τ*), ⋯, *X*(*t* + *dτ*)](*d* = 1, ⋯, 20). A plus sign represents an outlier.

The accuracy rate depending on the augmented units is shown in Fig 7. After 1000 combinations of three units were selected randomly, 122 out of those 1000 combinations were found for which the accuracy was increased by more than 10% through the augmentation. The horizontal axis of the figure represents the number of pseudo-units by augmenting data. The accuracy were confirmed to increase with the number of augmented units. The results imply that the use of time delays is an effective method of increasing the correct answer rate when only a few time series are obtained. However, the augmentation effect is limited for the combination of small number of units. It increases the correct answer rate, but insufficiently.

### Classification using multinomial logistic regression

**Fig 8:**
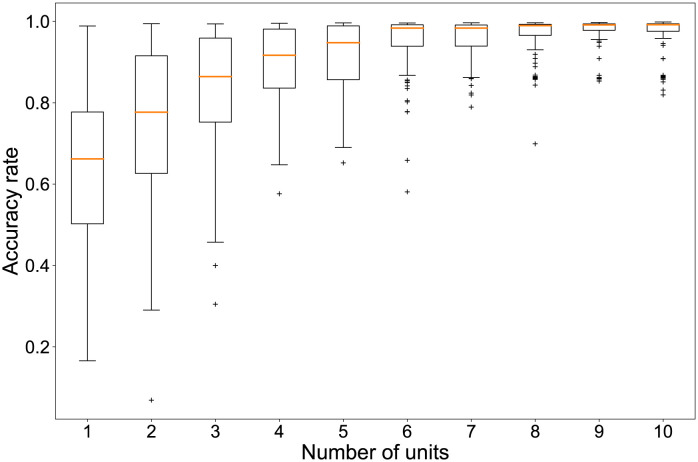
Accuracy rate of six-classification depending on the number of sampled units. Boxplots show the distribution of the percentage of correct answers for a randomly selected combination of reservoir units when using multinomial logistic regression. The vertical axis shows the percentage of correct answers. The horizontal axis shows the number of randomly selected units. For each number of units, we tried 1000 combinations of units. A plus sign denotes an outlier.

For the proposed method, the readout weights are trained using ridge regression. However, other regression methods are applicable if one assumes linearly separable dynamics. As one, we examine the effect of introducing nonlinearity to improve the classification accuracy. The result is presented in Fig 8. Using multinomial logistic regression, we observed the relation between the number of units and the percentage of correct answers when the readout weights were re-trained by increasing the number of reservoir units, as presented in Fig 3. The upper limit of the number of iterations to estimate the parameters for each variable was set to 1500.

The horizontal axis shows the number of reservoir units. The vertical axis shows the percentage of correct answers. Results show that the rate of correct answers is clearly higher when multinomial logistic regression is used for training, even with fewer units than those used for ridge regression. However, multinomial logistic regression required more time to estimate the regression coefficients as the number of units increased. Because of the introduction of nonlinearity, a trade-off was found between the longer computation time and the improved classification accuracy.

## Discussion

### Summary of results

For this study, we extracted feature points from movies of swaying plants and demonstrated classifying wind direction and speed using plants as naturally occurring physical sensors. Figs 2 and 4 show that we were able to classify about 99% of all test data into the correct class, even for 24-class classification with extended three wind speeds and eight directions.

The absolute value of the readout weights, as calculated using ridge regression with the extracted dynamics from the movie, is related to variation of the motion of the feature points. The coordinates of feature points corresponding to large absolute weights are all detected on leaves and stems with slight motion. The absolute weights corresponding to large motion tend to be small. The readout weights are presumed to reflect information such as the amplitude of time series of each coordinate and the relative positions between feature points. Therefore, the method enables classification with raw time series data. As a result, we demonstrated the possibility of using the dynamics extracted using the image processing technique as a reservoir unit.

We verified the number of reservoir units required for six-class classification. As shown in Fig 3, when the number of units was one, the correct answer rate was 16.7%, which was equal to the rate obtained when the classes were classified randomly. If one assumes the median correct answer rate when 1000 randomly selected combinations of reservoir units are used for classification, then at least eight reservoir units would be selected to classify more than 90% of the input data correctly. In this case, it was also confirmed that the percentage of correct answers dropped to about 60% when the units were not selected properly. Even if extracting feature points were not possible for the entire plant, the findings indicate that accurate classification was possible for some feature points. This result implies that computation based on the plants is robust against missing reservoir units.

Additionally, we confirmed the ability of generalization for the plant computing. Fig 5(f) shows that the plant remembers the direction of the wind in the readout weight. If one pattern out of six was not learned for *W*_*out*_, then the method can classify the not-learned pattern, at least for its wind direction. Therefore, oscillation of the plant responding to the same direction of wind is mutually similar for winds of different strengths. This finding implies that computation based on plants has some capability for generalization.

As shown in the figure included with S1 File, attractors were plotted for each class represented by different colors. Coordinates for the attractors are selected for three reservoir units that are selected randomly in the case of classification using 20 reservoir units. The transition among attractors is observed in the dynamics of three reservoir unit spaces over time. As a point of future difficulty to be assessed, the analysis of the reservoir states and readout weights leads to an estimation of physical structures such as the growth of plant branches and leaves.

The proposed approach’s adaptability warrants examination in relation to the dynamic nature of wind, encompassing both its speed and its continuous directional shifts. Furthermore, the criteria for an approach’s efficacy ought to be tailored to the specific botanical species under consideration. Although we have not yet undertaken a comprehensive exploration of a wide array of plant species, the present findings demonstrate the feasibility of estimating wind speed and direction for a subset of plant classifications. Importantly, these current results illuminate the practical implementation of computation harvesting and offer a potential framework for its validation. Future endeavors will entail investigation of additional plant species and varying wind conditions.

We posit that a key strength inherent to the proposed approach lies in its cost-effectiveness when compared to conventional anemometry techniques. This strength is attributed primarily to the utilization of object motion captured through cinematography as the core data source. The infrastructure for such cinematic data acquisition is pervasive within the real world, as exemplified by the prevalence of surveillance cameras, vehicular dashcams, and similar technologies.

### Comparison with conventional reservoir computing

The task examined for this study is not so difficult that the accuracy of classification was high. If the wind direction and speed were a matter of regression or prediction tasks, then the accuracy would not be sufficiently high. Moreover, the proposed method used only the linear combination of the motion of reservoir units on plants. If the task were more difficult than the current case, then the accuracy might be low only for use with linear regression. The conventional model of reservoir computing is built on a combination of affine and nonlinear transformations, which improve information processing capabilities [26]. Regarding the performance of reservoir computing which incorporates nonlinear and linear transformations, results demonstrated a trade-off between the increase in processing power and the decrease in storage capacity brought about by the introduction of nonlinearity to the activation functions of echo state networks [27]. It has also been confirmed that the use of time-delayed dynamical systems, which provides strong nonlinearity, gives excellent performance even for a single operation node [28]. More recently, auto reservoir computing has been proposed by which the reservoir units are embedded in the target systems with random neural networks attached to the output layer. It outperforms conventional reservoir models in prediction tasks [29]. From current computing methods, it has been observed that nonlinearity improves the classification performance shown in Fig 8. However, nonlinear transformation introduced into the computation can take some time. A trade-off exists between the performance accuracy and the necessary computational time.

As described in this paper, we demonstrated the task of classifying wind speed and direction using naturally occurring sensors from a movie, based on the “computation harvesting” concept. To elucidate, ‘computation’ is now construed as the extraction of alternative patterns of computational processes originating from the intricate dynamics inherent in the natural world. It is noteworthy that the inherent motion of plants is not to be regarded as a computational process per se. Computation harvesting entails entrustment of these processes to the natural dynamics, albeit with human intervention to complete the computational aspect. Numerous methods exist to extract computationally capable dynamics from natural phenomena. The desired computation can be considered in multiple ways. Under the framework of computation harvesting, we have described other tasks such as traffic volume prediction, based on actual traffic dynamics [30, 31]. This idea of computation harvesting is related closely to physical reservoir computing, but it differs fundamentally from the reservoir units. Naturally occurring phenomena indicate the dynamics. Therefore, the computational energy expenditure is quite low.

In contrast to earlier similar research endeavors, this study advocates for the utilization of natural phenomenon videos as a resource for reservoir computing. This approach is not confined solely to botanical subjects, but rather extends its applicability to any object exhibiting responsive motion to external stimuli. Its defining feature lies in its remarkably expansive scope of applicability. Another distinctive facet of this methodology is its capacity to be applied without the need for object-specific design, specifically un-designed, and relying solely on video imagery.

### Characteristics of computation harvesting

As characteristics of computation harvesting from nature dynamics, we consider the following properties: i) robustness against sensor failure, ii) generalizing capability, and iii) real-time response. The first two have been discussed in the preceding subsections. The real-time performance is an important benefit of the proposed framework. Because reservoir computing assumes that the number of learning weights is much smaller than RNN only for output weights, the computational time for reservoir computing is much shorter. Because computation harvesting is based on physical reservoir computing, it is suitable for real-time computing without the use of large amounts of computational resources.

In addition to the properties described above for computing, sensing dynamics in nature is useful for various purposes of computing. Results demonstrated that assessment of traffic dynamics is useful not only for predicting traffic volumes but also for prediction of temperature, which can be demonstrated using numerical simulations [32]. From this perspective, a sort of nature dynamics is useful for explaining other natural phenomena. This mode of dynamics is the influential feature of computation harvesting. In other words, it can be a means of finding unintended correspondences in real-world phenomena.

Finally, we discuss the scope of application of computation harvesting. For instance, the computational capability of natural phenomena in view of reservoir computing is evaluated using indices such as memory capacity, information processing capacity, and nonlinearity of systems. An important issue is quantifying the relation between these indices and the expected computational ability for harvested targets. Moreover, Echo state property (ESP) is available in the framework of reservoir computing. Therefore, the properties above can be verified as satisfied in the target for assessing the applicability of computation harvesting to natural dynamics. Although this avenue remains largely uncharted, we anticipate dedication of further attention to it as a future research endeavor. Furthermore, in the context of an experimental system, there exists some potential to manipulate the input variables, thereby enabling the calculation of memory capacity.

## Supporting information

S1 File Supporting figures and movies are in the supporting document. (PDF)
