# Supplementary material for: Computation harvesting from nature dynamics for predicting wind speed and direction
Source: PLoS One. 2023 Dec 14;18(12):e0295649. doi: 10.1371/journal.pone.0295649 (PMC10721085; doi:10.1371/journal.pone.0295649)
Supplement: S1 File — (PDF) [file pone.0295649.s001.pdf]

Supporting information for Computation harvesting from nature dynamics for predicting wind speed and direction

Takumi Aita[1], Hiroyasu Ando[2*], and Yuichi Katori[3, 4]

[1]Graduate School of Science and Technology, University of Tsukuba, Tsukuba, 305–8573, Japan

[2]Advanced Institute for Materials Research, Tohoku University, Sendai, 980–8577, Japan

[3]School of Systems Information Science, Future University of Hakodate, Hakodate, 041–8655, Japan

4Institute of Industrial Science, The University of Tokyo, Tokyo, 153–8505, Japan

*hiroyasu.ando.d1@tohoku.ac.jp

Supporting Figures

This file includes supporting figures appeared in the main text.

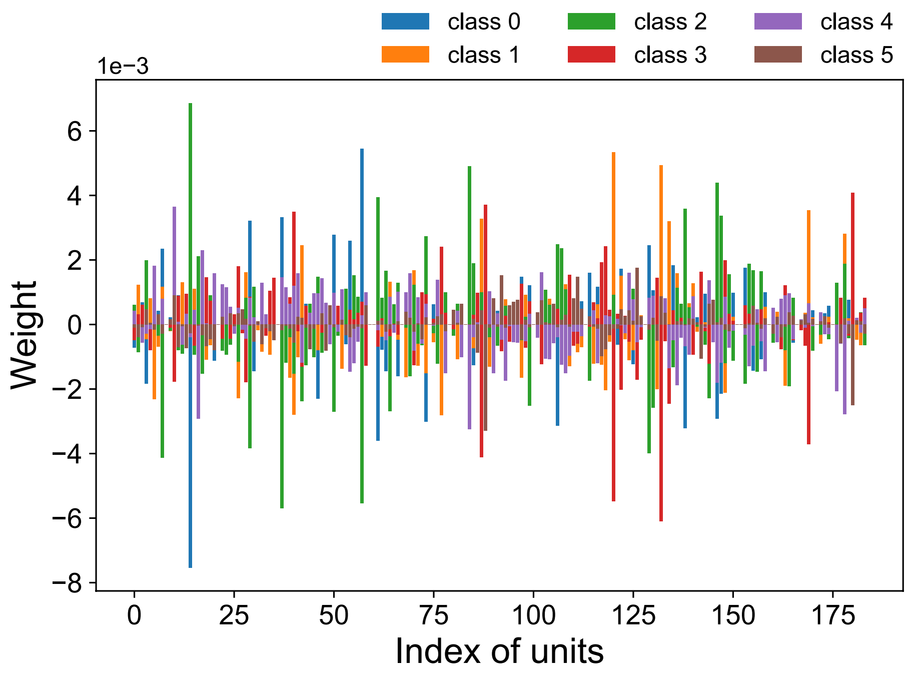

(a) Readout weights.

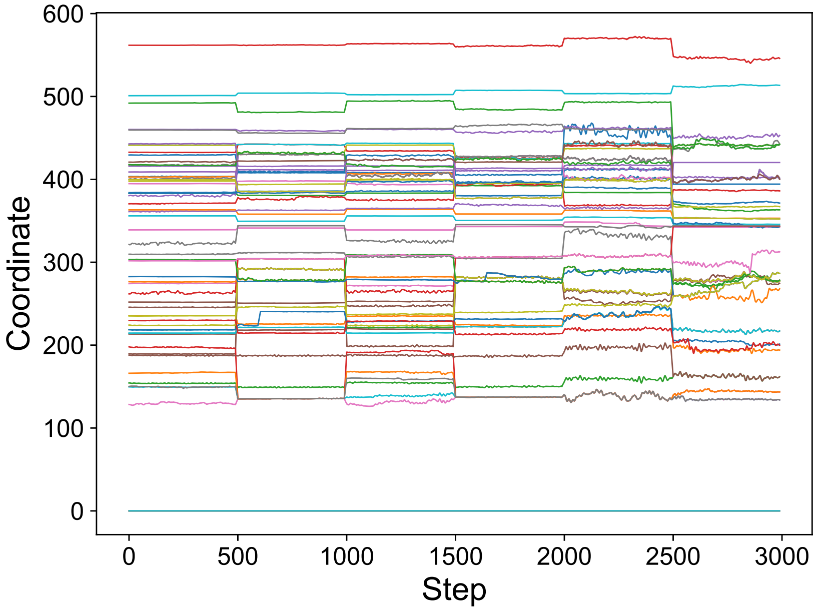

(b) Test data.

Supporting Figure 1. (a) Readout weights and (b) time series data of six-class classification. Supporting Fig. 1a shows W_{out} obtained using the Ridge regression. Weights corresponding to each of the six classes are depicted in different colours, e.g., the weight for class 0 is a blue bar. Supporting Fig. 1b shows time series data used to evaluate the percentages of correct answers, from unit 1 to unit 60, of all the detected reservoir units. The horizontal axis shows the time step. The vertical axis shows the x and y co-ordinate values.

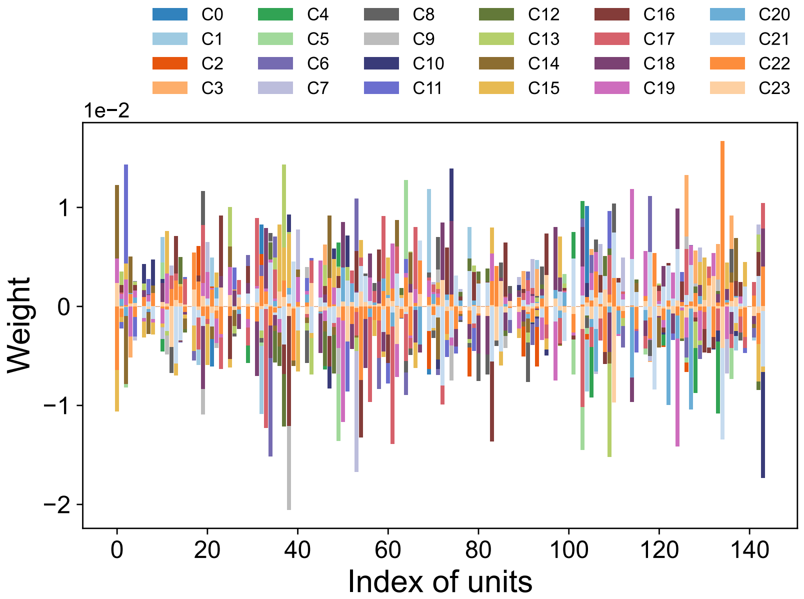

(a) Readout weights.

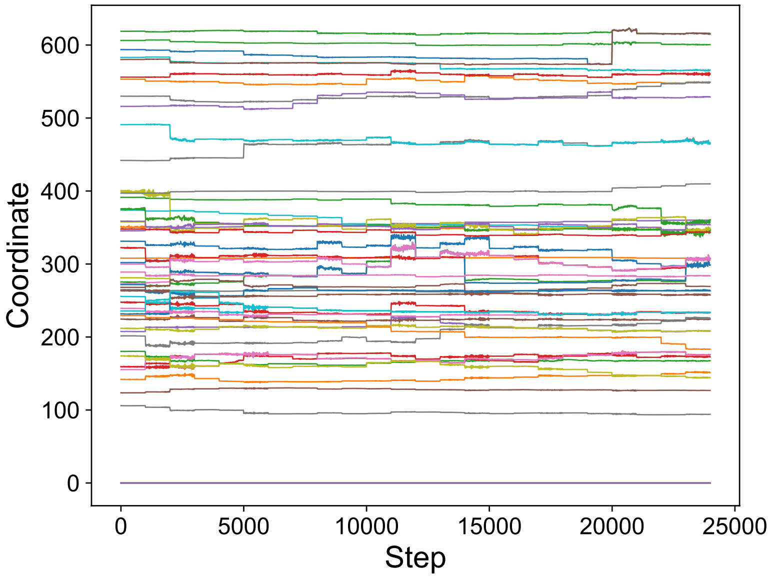

(b) Test data.

Supporting Figure 2. (a) Readout weights and (b) time series data of 24-class classification. Supporting Fig. 2a shows the W_{out} obtained by the Ridge regression. Weights corresponding to each of the 24 classes are shown in different colours. Supplorting Fig. 2b presents time series data used for evaluation of the correctness rate, from unit 1 to unit 60 of all the detected reservoir units. The horizontal axis shows the time step. The vertical axis shows the x and y co-ordinate values.

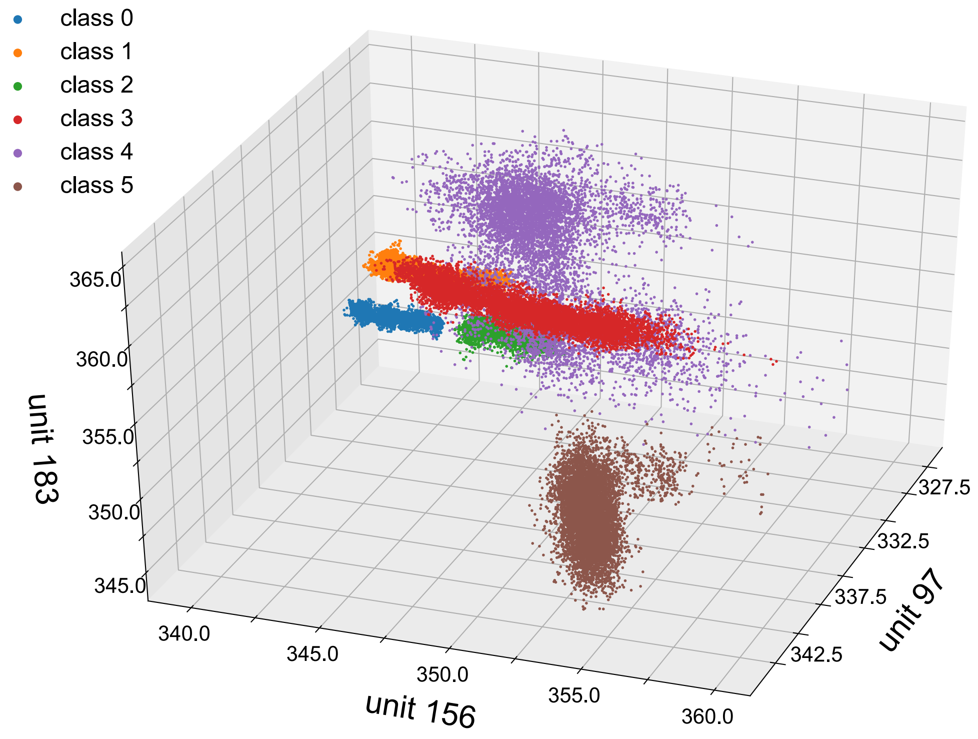

Supporting Figure 3. Distribution of attractors that correspond to the six classes. The three axes have unit identification numbers of 97, 156, and 183, respectively. The values of the axes are all y co-ordinates.

Supporting Movies:

The following links include movies analyzed in the manuscript.

Movie 1: Wind from the left and the strength is low.
https://www.dropbox.com/s/7o18x7nv05ef0jn/P2200783_M_L_1_max_pl.mp4?dl=0

Movie 2: Wind from the left and the strength is middle.
https://www.dropbox.com/s/ymfnmnz0wfszohy/P2200784_M_L_2_max_pl.mp4?dl=0

Movie 3: Wind from the left and the strength is high.
https://www.dropbox.com/s/76qzozqzd2sf8m8/P2200785_M_L_3_max_pl.mp4?dl=0

Movie 4: Wind from the right and the strength is low.

https://www.dropbox.com/s/ovq66c8ctsud0ay/P2200786_M_R_1_max_pl.mp4?dl=0

Movie 5: Wind from the right and the strength is middle.
https://www.dropbox.com/s/f4uwcilil74ijh3/P2200787_M_R_2_max_pl.mp4?dl=0

Movie 6: Wind from the right and the strength is high.
https://www.dropbox.com/s/a0ese1ymb6l02ii/P2200788_M_R_3_max_pl.mp4?dl=0
